# TRPM4-mediated control of FcεRI-evoked Ca^2+^ elevation comprises enhanced plasmalemmal trafficking of TRPM4 channels in connective tissue type mast cells

**DOI:** 10.1038/srep32981

**Published:** 2016-09-14

**Authors:** Torben Rixecker, Ilka Mathar, Rebekka Medert, Stefanie Mannebach, Alexander Pfeifer, Peter Lipp, Volodymyr Tsvilovskyy, Marc Freichel

**Affiliations:** 1 Pharmakologisches Institut, Ruprecht-Karls-Universität Heidelberg, 69120 Heidelberg, Germany; 2 Experimentelle und Klinische Pharmakologie und Toxikologie, Universität des Saarlandes, 66421 Homburg, Germany; 3 Institute of Pharmacology and Toxicology, University Hospital Bonn, University of Bonn, 53127 Bonn, Germany; 4 Institut für Molekulare Zellbiologie Universität des Saarlandes, 66421 Homburg, Germany

## Abstract

TRPM4 proteins form Ca^2+^-activated non selective cation (CAN) channels that affect transmembrane Ca^2+^-influx by determining the membrane potential. Tight control of the intracellular Ca^2+^ concentration is essential for mast cell responses. In this study, we analyzed the expression of TRPM4 in peritoneal mast cells (PCMC) as a model for connective tissue type mast cells with respect to FcεRI-evoked calcium changes and the subcellular localization of fluorescently labeled TRPM4 using two viral transduction systems before and following antigen stimulation. Our results show that TRPM4 is expressed in PCMCs, is an essential constituent of the endogenous CAN channels in PCMCs and regulates antigen-evoked increases in intracellular calcium that are significantly enhanced in TRPM4-deficient PCMCs. Compared to PCMCs analyzed before antigen stimulation, the cells depict a substantially increased localization of TRPM4 proteins towards the plasma membrane after FcεRI stimulation. Thus, TRPM4 functions as a limiting factor for antigen evoked calcium rise in connective tissue type mast cells and concurrent translocation of TRPM4 into the plasma membrane is part of this mechanism.

Mast cell activation triggered by various stimuli involves an increase in cytosolic calcium inducing a multitude of cellular responses including the release of preformed mediators following degranulation, production of eicosanoides, synthesis of cytokines as well as cell migration. Tight control of the intracellular Ca^2+^ concentration triggered by numerous Ca^2+^ mobilizing mast cell activators is essential for mast cell responses and the importance of extracellular Ca^2+^ as a requirement for release of histamine was already shown more than 40 years ago^1^,^2^. TRP channels can directly contribute to Ca^2+^ influx via the plasma membrane as constituents of Ca^2+^ conducting channel complexes or indirectly by shifting the membrane potential and regulation of the driving force for Ca^2+^ entry through independent Ca^2+^ entry channels in many cell types including mast cells^3^. In the light of the lack of agonists and/or antagonists with sufficient specificity for most members of the TRP channel family, the analysis of the contribution of these channels to above mentioned processes involved in mast cell activation has so far been mainly studied using small molecule inhibitors in human mast cells or mast cell lines^4^, using knock-down approaches by RNA interference^5^,^6^ or employing bone marrow derived mast cells (BMMCs) isolated from knockout mouse lines^7^,^8^,^9^,^10^. However, BMMCs differ in their characteristics and activation mechanisms from tissue mast cells in various aspects^11^, e.g. BMMCs cannot be activated by IgG immune complexes and the release of inflammatory mediators by degranulation is much lower^12^. Mast cells cultured from the peritoneal lavage (PCMCs) represent a valuable mast cell model that resembles connective tissue type mast cells (CTMC) which predominate e.g. in the skin and are activated during the development of cutaneous anaphylaxis^13^. Cultures of PCMCs were initially described by Enerbäck *et al*. in 1970^14^ and were later developed further^12^. In PCMCs stimulation of the high-affinity Fc receptor for IgE (FcεRI) and beta hexosaminidase release is increased eightfold and hundredfold, respectively, compared to BMMCs.

Recently, we and others showed that TRPM4 acts as a calcium-activated cation channel that limits calcium entry via CRAC channels through membrane depolarization in Jurkat T cells, BMMCs and dendritic cells^8^,^15^,^16^. Thereby, TRPM4 channels control the release of inflammatory mediators such as histamine, leukotrienes, interleukines (IL-2, IL-6) and TNFα. In BMMCs, Ca^2+^ -activated and TRPM4-mediated cation currents developed with a variable delay of more than 20 seconds after obtaining whole cell configuration and are characterized by a continuous increase over several minutes thereafter^8^. Furthermore, work in pancreatic beta (INS-1) and smooth muscle (A7r5) cell lines suggested a translocation of TRPM4 proteins from intracellular organelles towards the plasma membrane contributing to the incremental increase of TRPM4 current density^17^,^18^. In these experiments, TRPM4 channels were stimulated by elevation of cytosolic calcium or by protein kinase C (PKC) activators, but evidence for receptor-operated translocation of TRPM4 proteins, particularly in primary mast cells, is still lacking.

In the present study, we aimed to analyze the expression of TRPM4 in peritoneal mast cells and their functional relevance for FcεRI-evoked calcium rise in PCMCs. Additionally, we tested different transduction methods in PCMCs to visualize TRPM4 proteins in their native environment using fluorescently labeled proteins and confocal microscopy to investigate whether translocation of TRPM4 proteins towards the plasma membrane can be identified in these connective tissue type mast cell model before and after allergen stimulation. TRPM4 was found to be co-expressed with its structurally most related family member TRPM5 in this type of mast cells. Our results indicate that TRPM4 is functionally relevant as a limiting factor for antigen-evoked calcium rise and that concurrent translocation of TRPM4 into the plasma membrane is part of this mechanism that limits mast cell activation in tissue mast cells.

## Results

### Detection of Trpm4 transcripts in peritoneal mast cells

**Figure 1:**
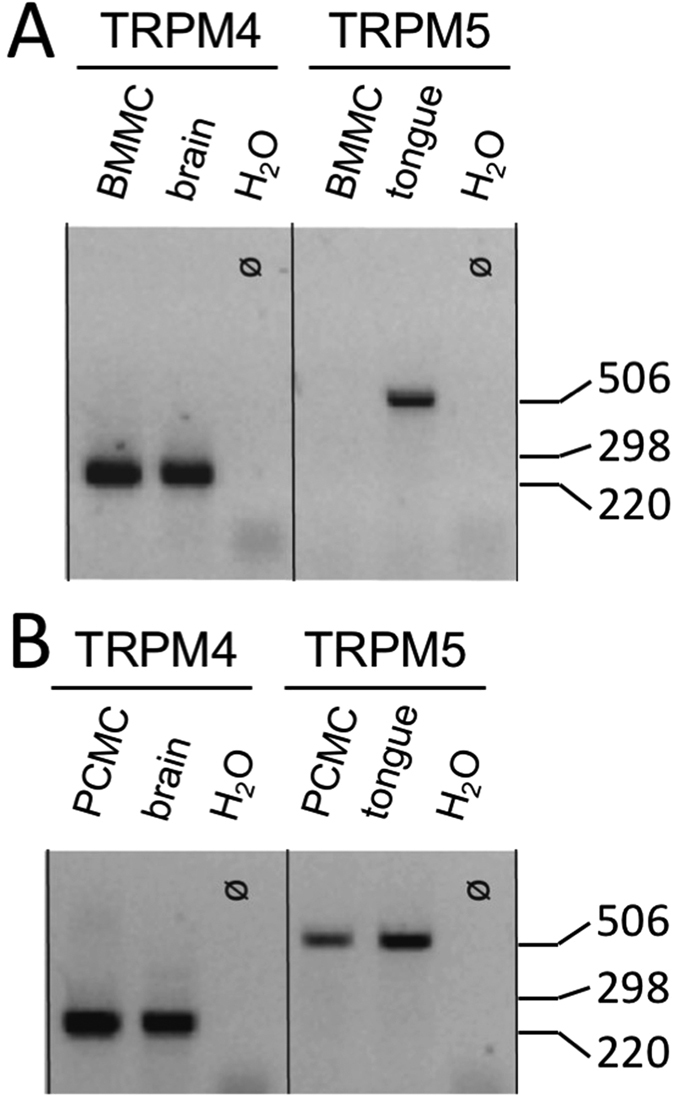
Amplification of Trpm4 (246 bp) and Trpm5 (484 bp) transcripts from RNA of BMMCs (**A**) and PCMCs (**B**) by RT-PCR. Analysis was performed on an 2% agarose gel. RNA from mouse brain (Trpm4) or mouse tongue (Trpm5) was used as control for the primer combinations. Amplification of HPRT transcripts was positive for all samples analysed (not shown). Similiar results were obtained in n = 3 independent preparations from both types of mast cells. 1 kb ladder was used as a size marker.

We compared the expression of TRPM4 transcripts in bone marrow derived mast cells (BMMC) (Fig. 1A) and in peritoneal mast cells as a model for connective tissue type mast cells (Fig. 1B). TRPM4 as well as TRPM5 transcripts were detected consistently in three independent PCMC cultures (Fig. 1B) whereas only TRPM4, but not TRPM5, could be amplified from bone marrow derived mast cells (Fig. 1A). These results raised the question whether inactivation of Trpm4 in PCMCs alters FcεRI-induced Ca^2+^ elevation or whether TRPM5 could compensate the function in the absence of TRPM4 in this type of mast cells following FcεRI stimulation.

### Inactivation of TRPM4 leads to enhanced FcεRI-evoked Ca^2+^ elevation in peritoneal mast cells

**Figure 2:**
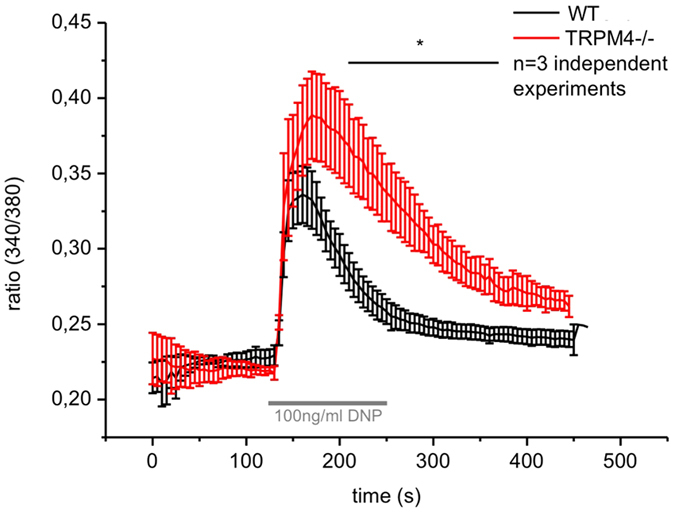
Inactivation of TRPM4 leads to enhanced FcεRI-evoked Ca^2+^ rise in peritoneal mast cells (PCMC). Mean traces for the time-dependent change in [Ca^2+^]_i_ in Fura-2-loaded Trpm4^+/+^ and Trpm4^–/–^ PCMCs are presented as the ratio of fluorescence at 340 nm to that at 380 nm (340/380). n = 3 independent experiments with at least 50 cells per single measurement were pooled for the analysis. Cells were incubated over night with 300 ng/ml anti-DNP-IgE and stimulated with 100 ng/ml DNP at the indicated time period.

To test the functional relevance of TRPM4 in this connective tissue mast cell model, PCMCs were incubated with anti-DNP IgE over night and after recording the baseline calcium concentration the antigen (DNP, 100 ng/ml) was applied. Similar to BMMCs, where TRPM5 expression was not detected^8^, we found that upon deletion of TRPM4 FcεRI-evoked calcium transients are significantly enhanced in PCMCs (Fig. 2). Specifically, the amplitude of antigen-evoked Ca^2+^ rise is increased (WT 0.108 ± 0.017, Trpm4−/− 0.186 ± 0.013, n = 3, p < 0.05) and the time to 50% decay (WT 53 ± 5 s, Trpm4−/− 106 ± 14 s, n = 3, p < 0.05) is prolonged in Trpm4-deficient PCMCs indicating that the loss of TRPM4 could not be compensated by other Ca^2+^ -activated cation channels including TRPM5.

### TRPM4-EYFP expression in PCMC before and after FcεRI-stimulation using SFV transduction vectors

Analyses of TRPM4 function in cell lines derived from pancreatic beta cells or vascular smooth muscle cells indicated that stimulation of TRPM4 channel activity by elevation of intracellular Ca^2+^ concentration using ionomycin or by activation of PKC using phorbol 12-myristate 13-acetate (PMA) evoked a higher abundance of TRPM4 in the plasma membrane^17^,^18^. To test whether TRPM4 translocation also occurs in primary mast cells particularly following native mast cell stimulation (i.e. after antigen application), we studied the distribution of fluorescently labeled TRPM4 (TRPM4-EYFP) in PCMCs before and after antigen stimulation. Since primary PCMCs are difficult to transfect we used two independent viral transduction systems, SFV and lentivurses (see below). PCMCs were successfully transduced with TRPM4-EYFP-SFV at a transduction effiency of 9,3% (Suppl. Fig. 1A,B). TRPM4-EYFP fluorescence in PCMC was observed from 6 hours (data not shown) after transduction onwards, but the time point 10 h after transduction was most appropriate for localisation studies with respect to fluorescence intensity and cell viability.

**Figure 3:**
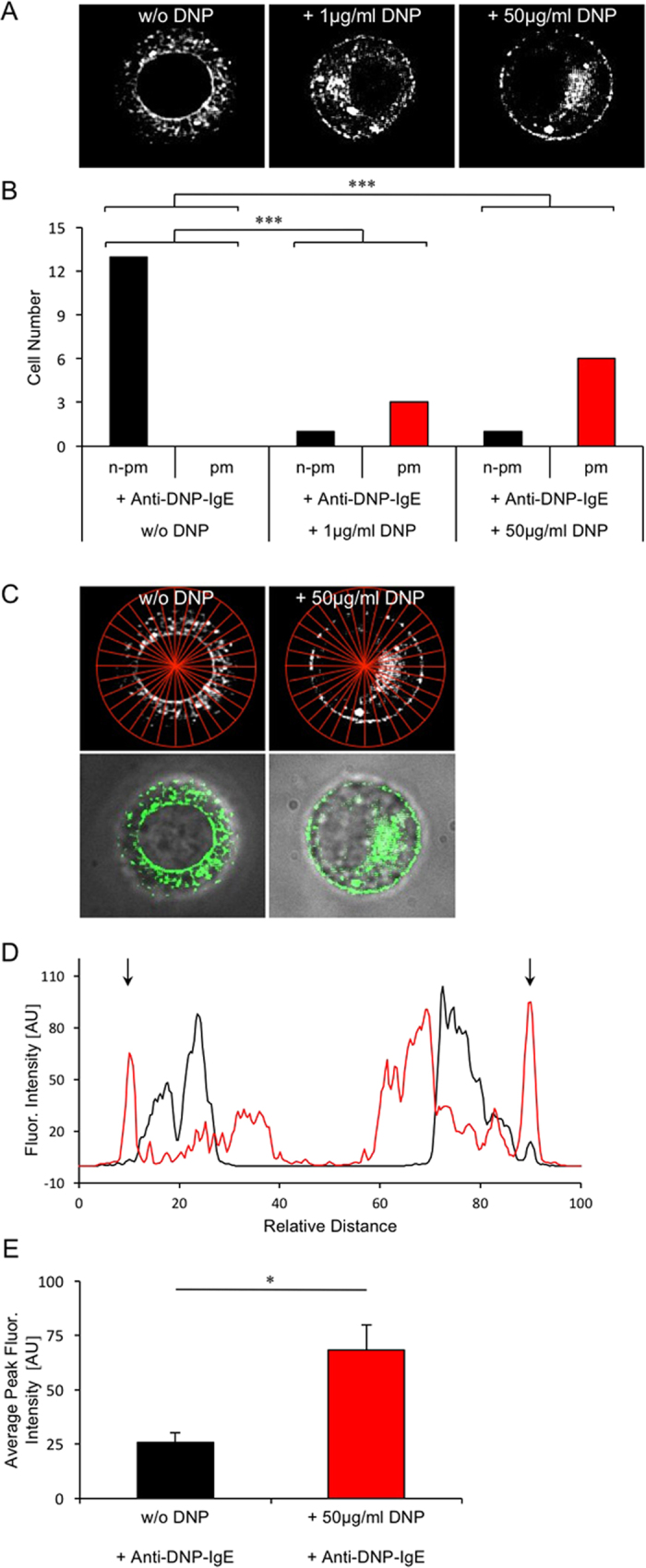
Fcε-RI stimulation induces TRPM4-EYFP translocation to the plasma membrane in PCMCs following Semliki Forest Virus mediated transduction. (**A**) Mast cells with substantial plasma membranous localization (pm) of TRPM4-EYFP and with non-plasma membranous (n-pm) TRPM4-EYFP localisation in PCMCs before (left panel: w/o DNP) and 20 to 30 minutes after DNP-stimulation (middle and right panel, DNP concentration indicated). All cells were incubated with 600 ng/ml anti-DNP-IgE and plated on 0.001% PLL coated coverslides 15 min before microscopy. (**B**) Bar graphs show the number of cells assigned to whether TRPM4-YFP could be identified in the plasma membrane (pm) or not (n-pm). p < 0.01 (Fisher’s exact test) for the camparison of control (w/o DNP stimulation, n = 5) and Fcε-RI stimulated (1 μg/ml DNP, n = 2 and 50 μg/ml DNP, n = 3, respectively). Fluorescence microscopy was performed using a Nikon E600 microscope with a QLC-100 scan head and a 100x water objective. Fluorescence was measured 10 h after cell transduction with TRPM4-EYFP SFV. (**C**) Analysis of fluorescence intensity distribution by multi plot analysis using 16 cell lines of PCMCs without FcεRI stimulation and with addition of 50 μg/ml DNP as shown in A (upper panel) and overlay of a confocal image of TRPM4-EYFP with phase contrast (lower panel). (**D**) Average fluorescence intensity profile of 16 lines as shown in C using multi plot analysis for one exemplary cell without (black trace) and one exemplary cell after FcεRI stimulation (50 μg/ml DNP, red trace), respectively. AU: arbitrary units; relative distance: distance in relation to the corresponding first and last fluorescence intensity peak of the averaged fluorescence intensity profile. (**E**) Comparison of average edging peak fluorescence intensity of all cells without (n = 13) and with FcεRI stimulation with 50 μg/ml DNP (n = 7). Shown are the means and the standard errors for each condition (*p < 0.05; T-test for independent samples).

To obtain stronger mast cell adherence we initially used Poly-L-lysin coating of coverslips at a high concentration (0.01% PLL, PLL_high_) which by itself resulted in a time-dependent translocation of TRPM4-EYFP proteins from intracellular organelles towards the plasma membrane without additional stimulation (Supplementary Fig. 2A,B), supporting the evidence of TRPM4-channel translocation upon mast cell stimulation since PLL has been described as a potent mast cell stimulant in previous work^19^,^20^. Since this PLL_high_ protocol did interfere with additional agonist evoked changes in TRPM4-EYFP translocations, a PLL_low_ protocol, in which cover slips were pretreated with 0.001% PLL, was used in all following experiments. To investigate potential changes in subcellular localization of TRPM4-EYFP proteins, PCMCs were first transduced with a TRPM4-EYFP construct using Semliki Forest virus particles. Pretreatment with IgE antibodies directed against the allergen (DNP) was carried out the night before and microscopic analysis of TRPM4-EYFP localization was carried out 10 hours following SFV transduction. All cells without DNP incubation mainly showed TRPM4-EYFP fluorescence in intracellular organelles, and TRPM4-EYFP was faintly detectable in the plasma membrane. In contrast, stimulation with DNP for 20 to 30 minutes resulted in a dose-dependent translocation of TRPM4-EYFP to the plasma membrane as the fraction of cells with substantial plasma membranous localization of TRPM4 increased significantly in DNP-treated PCMCs (Fig. 3A,B). To quantify plasma membranous TRPM4-EYFP expression, the distributon of mean YFP fluorescence was analysed in an x-y plot of 16 random lines per cell (Fig. 3C). Comparison of the edging peak fluorescence intensity of all cells without (n = 13) and with FcεRI-stimulation (50 μg/ml DNP, n = 7) revealed a significant increase in plasma membranous TRPM4-EYFP expression (Fig. 3D,E) suggesting translocation of TRPM4 proteins from intracellular organelles towards the plasma membrane following antigen stimulation.

### TRPM4-EYFP expression in PCMC upon FcεR-stimulation using lentiviral transduction vectors

**Figure 4:**
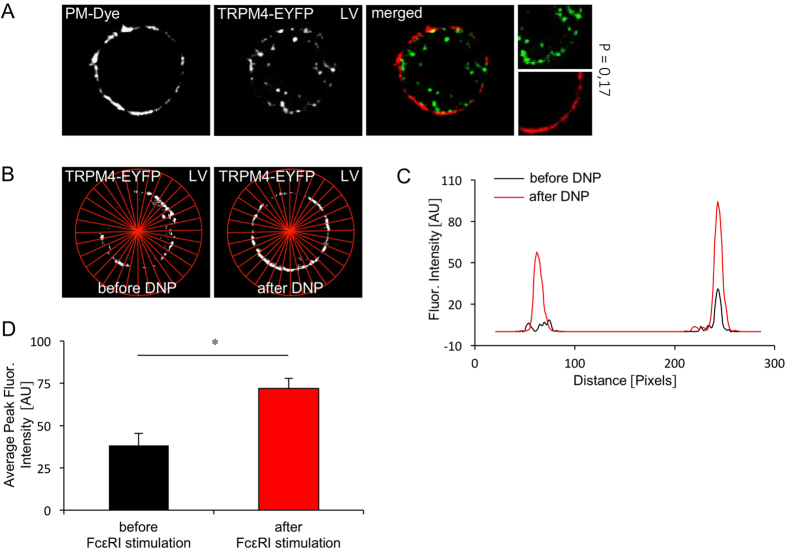
Fcε-RI stimulation induces TRPM4-EYFP translocation to the plasma membrane in PCMCs following lentiviral transduction. (**A**) TRPM4-EYFP expression after lentiviral transduction in non stimulated PCMC (PLL _low_). The plasmamembrane is visualized using the cell mask deep red stain (Invitrogen). (P = Pearson’s coefficient, calculated for the presented image section). (**B**) TRPM4-EYFP expression in the same cell before and 20 to 30 min after FcεRI stimulation. PCMC’s were stimulated with 1 μg/ml DNP. The cells were incubated with 600 ng/ml anti-DNP-IgE for 13 h and plated on 0.001% PLL coated coverslides 15 min before microscopy. Analysis of fluorescence intensity distribution by multi plot analysis using 16 cell lines before and after FcεRI stimulation. (**C**) Average fluorescence intensity profile of 16 lines as shown in B using multi plot analysis for one exemplary cell before and after FcεRI stimulation. (AU = arbitrary units). (**D**) Average peak fluorescence intensity as shown in B, C of 4 independent masurements (4 cells) before and after FcεRI stimulation. Shown are the means and the standard errors for each condition. (*p < 0.05; T-test for dependent samples, n = 4).

To evaluate these results based on SFV transduction with more constant expression levels, TRPM4-EYFP lentiviral vectors were prepared. The TRPM4-EYFP lentivirus successfully transduced PCMC at a transduction efficiency of 3,4% (Suppl. Fig. 1). Analysis of TRPM4-EYFP localisation by confocal microscopy (Fig. 4) was performed between day 14 when calcium imaging experiments were started until day 28 after cell transduction. The proportion of TRPM4-EYFP in intracellular vesicles under basal conditions was less prominent following lentiral transduction (Fig. 4A) compared to transduction via SFV (Fig. 3A). Costaining of the TRPM4-EYFP fluorescence with the cell mask deep red plasma membrane stain indicated that only a low proportion of TRPM4 can be detected in the plasma membrane under basal conditions (Fig. 4A). However, for further colocalisation and translocation analysis following FcεRI-stimulation, the cell mask plasma membrane stain turned out to be an inappropriate tool since stimulated PCMCs showed plasma membrane extrusions (see Suppl. Fig. 2C) which are known to be evoked following calcium-dependent mast cell activation^21^, leading to underestimated plasma membrane expression levels of TRPM4-EYFP. Thus, experiments adressing TRPM4-EYFP translocation triggered by FcεR I-stimulation after lentiviral transduction were again performed according to the experiments following SFV based transduction by confocal microscopy before and after DNP stimulation. Localisation in the plasma membrane was quantified by YFP fluorescence distribution in an x-y plot analysis of 16 random lines per cell (Fig. 4B,C). In 4 independent measurements a significant increase of the peak fluorescence at the edge of the cells was observed after DNP stimulation strongly suggesting that FcεR-stimulation evokes an increase in the amount of plasmalemmal TRPM4 channel proteins (Fig. 4D).

### Time course of endogenous TRPM4-mediated CAN-Currents in peritoneal mast cells

**Figure 5:**
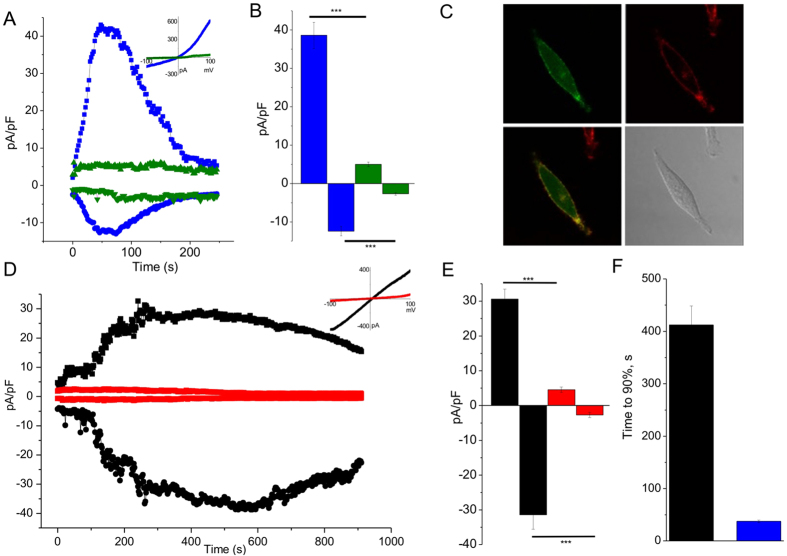
Time course of CAN currents of endogenous TRPM4-containing channels in PCMCs and heterologously expressed TRPM4 channels. (**A)** Time course of the whole-cell current amplitude at −80 mV and +80 mV in TRPM4-YFP positive (blue) and TRPM4-YFP negative (green) HEK cells dialyzed with a pipette solution containing 10 M Ca^2+^. (Insert). Current-voltage relationships of corresponding currents at time points of their maximal amplitude. (**B)** Mean current densities of currents at +80 mV (positive values) and −80 mV (negative values) obtained in an experiment as described in A. Data represent 5 and 3 experiments TRPM4-YFP positive and negative cells corresponding. *P* < 0.001. (**C**) Confocal images of TRPM4-YFP positive HEK cells: YFP fluorescence (left top), cell mask fluorescence (right top), overlay of previous images (left bottom), DIC image (right bottom). (**D**) Time course of the whole-cell current amplitude at −80 mV and +80 mV in *Trpm4*+/+ PMCs (black) and *Trpm4*−/− PMCs (red) dialyzed with a pipette solution containing 10 M Ca^2+^. (Insert). Current-voltage relationships of corresponding currents at time points of their maximal amplitude. (**E**) Mean current densities of currents at +80 mV (positive values) and −80 mV (negative values) obtained in an experiment as described in D. Data represent 5 experiments for each condition. *P* < 0.001. (**F**) Mean values of the “time to 90% of maximum” of outward currents at +80 mV in HEK cells (black; n = 5; corresponds to experiments described in A,B) and in PMCs (blue; n = 5; corresponds to experiments described in D,E).

The development of endogenous TRPM4-mediated currents in BMMCs^8^ was much slower compared to that of heterologously expressed TRPM4 channels^22^,^23^ and a second phase of activation after a variable delay in the range of several minutes after formation of whole cell configuration was reported in some cell systems, which might be due to recruitment of TRPM4 to the plasma membrane^24^. Since we found a significant increase in the translocation of EYFP-labeled TRPM4 towards the plasma membrane following FcεR I-stimulation in PCMCs we aimed to study the kinetics of endogenous TRPM4-mediated cation currents in these cells and compared it with currents obtained following heterologous expression of TRPM4-EYFP. HEK293 cell stably transduced with the lentivirus encoding TRPM4-EYFP (HEK-TRPM4-YFP-LV) showed a fast current development after intracellular perfusion with a pipette solution containing 10 μM [Ca^2+^]_i_. The time to 90% of the peak was below 50 seconds (Fig. 5A,B,F) which is very similar to currents that were obtained with TRPM4 proteins lacking the YFP tag in other studies^22^,^23^ and indicates functional efficiency of the TRPM4-EYFP construct. However, considerable differences in the biophysical characteristics between the CAN currents evoked by heterologous expression of TRPM4 and the endogenous TRPM4-mediated currents in PCMCs become obvious from the correspondning I–V curves (Fig. 5B,D). Overlay of the TRPM4-EYFP derived fluorescence with DIC images of the same HEK-TRPM4-YFP-LV cells shows that TRPM4-EYFP proteins are readily detectable in the plasma membrane without cell stimulation. In contrast, the TRPM4-mediated plasma membrane current of WT-PCMC rises over several minutes after elevation of cytosolic free Ca^2+^ concentration (Fig. 5D,E) reaching 90% of its maximum only after 400 sec (Fig. 5F). The slow incremental augmentation of TRPM4-mediated CAN currents in PCMC, which are not detectable in Trpm4−/− PCMCs (Fig. 5D,E), and the increase of the abundance of TRPM4-EYP proteins at the plasma membrane following FcεR I-stimulation (Figs 3 and 4) strongly suggests that translocation of TRPM4 into the plasma membrane is part of the mechanism how TRPM4 channels operate as a limiting factor for antigen evoked calcium rise in connective tisse type mast cells.

## Discussion

In this study we analyzed the functional role of TRPM4 proteins as essential constituents of CAN channels and for FcεRI-evoked calcium transients in PCMCs as a model for connective tissue type mast cells and addressed the question of receptor stimulation-evoked concurrent translocation of these channel proteins towards the plasma membrane following receptor stimulation. Experiments in a passive cutaneous anaphylaxis model already showed the relevance of TRPM4 for activation of connective tissue type mast cells. Antigen evoked fluid extravasation triggered by FcεRI-activation of cutaneous mast cells in the vicinity of blood vessels is increased in TRPM4 deficient mice^8^. However, antigen evoked changes in calcium homeostasis in connective tissue type mast cells have not been analyzed so far. In addition, the mechanisms leading to a delayed development of TRPM4-mediated cation currents in mast cells raised the question whether receptor evoked trafficking of TRPM4 channel proteins might contribute to this process.

Fluorimetric measurements showed that FcεRI-evoked Ca^2+^ transients were significantly increased in TRPM4-deficient peritoneal mast cells compared to wild type controls. These results indicate that TRPM4 is an essential regulator of antigen evoked calcium rises in connective tissue type mast cells. Accordingly, TRPM4 is likely to control Ca^2+^ dependent processes during mast cell activation such as degranulation and release of inflammatory mediators like in BMMCs to limit the development of cutaneous anaphylaxis^8^. In our RT-PCR results we found that TRPM4 and TRPM5 are coexpressed in peritoneal mast cells, although a contamination from other cell types cannot be excluded ultimately with 98.5 to 99.5% purity of the PCMC cultures. Nevertheless, a functional role of TRPM5 remains to be identified in these cells. Despite this the lack of TRPM4 cannot be compensated by TRPM5 or other ion channels since FcεRI-evoked Ca^2+^ transients are significantly increased in PCMCs lacking TRPM4. This could not be expected or extrapolated from previous studies using BMMCs in which TRPM4, but not TRPM5 could be identified. In most cell types the expression of TRPM4 and TRPM5 is not overlapping^24^,^25^. TRPM5 is expressed and functionally relevant in distinct cell types such as taste receptor cells where TRPM5 is the final element in the signaling cascade triggered by bitter, sweet or umami taste molecules^26^,^27^. TRPM5 is also found in pancreatic islets where it finetunes insulin secretion^26^. In contrast, inactivation of TRPM4 does not affect glucose evoked increase in insulin secretion^8^. Obviously, both channel proteins, which are essential constituants of calcium activated cation channels, have distinct functional roles even if they are coexpressed in individual cell types.

In the present study we showed for the first time that in primary mast cells TRPM4 proteins translocate towards the plasma membrane following a naturally occuring stimulus, i.e. FcεRI stimulation by an antigen. For visualization of TRPM4 proteins in PCMC we used TRPM4-EYFP fusion proteins whose expression was mediated by viral transduction using two different viral vector systems because for immunocytochemical detection of TRPM4 cells required prior permeabilisation that substantially impaired plasma membrane detection of the protein. Prior experience with experiments using exogeneous protein expression in primary mast cells was very limited in the past and mainly based on nucleofection techniques^28^, while viral transduction in PCMCs was not reported. In human lung mast cells (HLMC) adenoviral vectors were succesfully used for transduction^29^, but in our hands for PCMCs application of adenoviral vectors encoding a fluorescent reporter in PCMCs did not reveal positive cells (data not shown). Nucleofection represents a transient expression system and evokes morphologically detectable cell damage in a large proportion of treated cells. We therefore seeked for alternatives for our subcellular localization experiments in these cells. Although the transduction efficiency of PCMCs using two different viral expression vectors was rather low (<10%), no changes in mast cell morphology were detected even in the rapidly expressing SFV system known for very fast and strong expression (see DIC images in Suppl. Fig. 1B). The main disadvantage using the SFV transduction system is the limited time window for conducting the experiments resulting from the very rapid transgene overexpression. Nevertheless, in comparison to the lentivirus transduction the SFV mediated expression conducted a higher fluorescence intensity in our hands. Otherwise, the former led to lower, more physiological expression levels and allowed functional analysis of the transduced transgene over a much longer time period^30^. Overlay of TRPM4-EYFP and phase contrast images indicated an increased abundance of TRPM4-EYFP at the plasma membrane following antigen stimulation using both transduction systems. Although the spatial resolution of confocal microscopy does not allow to localize the fluorophore in the membane itself these results implicate recruitement of TRPM4 proteins into the plasma membrane upon FcεRI-stimulation.

To correlate the translocation of TRPM4 channel proteins into the plasma membrane with the development of TRPM4 currents in PCMC we investigated the time course CAN currents in these cells in comparison to heterologously expressed TRPM4 channels. Similarly as shown before for untagged TRPM4 proteins^22^,^23^, overexpression of TRPM4-EYFP in HEK293 cells leads to a fast activation of the TRPM4-mediated CAN currents within the first minute after cytosolic Ca^2+^ application whereas endogenous TRPM4-meditaed CAN currents in PCMCs approach their peak not before 7 minutes after break in. Therefore it is tempting to speculate that mast cell stimulation evokes a recruitment of TRPM4 channel proteins from intracellular, vesicular organelles to the plasma membrane which may explain the observed latency of evolving TRPM4-mediated cation currents in PCMC. The Ca^2+^ -activated currents that are lacking in corrresponding cells from Trpm4−/− mice exhibit a linear IV-relationship. TRPM4-dependent currents with very similar I-V relationsship were already demonstrated in bone marrow derived mast cells (BMMC)^8^. Interestingly, Barbet *et al*.^15^ showed an outwardly rectified, Ca^2+^ -activated current in dendritic cells, which was absent in Trpm4−/− cells, and currents with similar characteristics were obtained in bone marrow-derived macrophages^31^. The reason for these differences in the I-V relationship are not solved until now, but might be the result of a disproportion of PIP_2_ and TRPM4 levels in the plasma membrane of the particular cell system as discussed since PIP_2_ shifts the voltage dependence of activation towards negative potentials and strongly increases the open probability at a physiological membrane potential^24^,^32^,^33^.

Translocation of TRPM4 to the plasma membrane was reported previously with stimuli that are generated downstream of the activation of plasmalemmal receptors such as the FcεRI. Analyses of TRPM4 function in cell lines derived from pancreatic beta cells or vascular smooth muscle cells indicated that stimulation of TRPM4 channel activity by elevation of intracellular Ca^2+^ concentration using ionomycin or by activation of PKC using phorbol 12-myristate 13-acetate (PMA) evoked a higher abundance of TRPM4 in the plasma membrane detected by microscopic approaches including TIRF microscopy which allows the identification of fluorescently labeled proteins in and in close vicinity to the plasma membrane^17^,^18^. In continous recordings of TRPM4-EYFP fluorescence in TIRF experiments a significant increase of TRPM4 translocation to the membrane was reported ten minutes after PMA stimulation^18^. Our analysis in a model of connective tissue type mast cells showed TRPM4-YFP translocation towards the plasma membrane 20 minutes after FcεRI-stimulation in PCMCs while the time course of electrophysiological recordings suggested that the peak of TRPM4-mediated CAN currents is attained not later then 10 minutes. Such incremental increase in CAN current density as an indicator of channel translocation was also used in a cell line derived from pancreatic beta cells^17^ and represents a more sensitive technique compared to currently available microscopic approaches, but current density analysis can be perturbed by other processes enhancing or inhibiting channel function independent of the channel protein translocation to the membrane. Overall, our results support the concept that TRPM4 trafficking from intracellular vesicles to the plasma membrane increases the number of available channel proteins mediating cation entry and that this process contributes to the continous increase in current density in addition to direct Ca^2+^ -dependent modifications of TRPM4 proteins as part of CAN channels already residing in the PM independent of or prior to agonist stimulation. Furthermore, our results extend the relevance of previous studies to a model of connective tissue type mast cells following stimulation with a (patho)physiological agonist.

Translocation of TRP channel proteins other then TRPM4 following receptor stimulation was also reported for other agonists and in other cell types. Growth factor induced translocation of TRPC5 channels to the plasma membrane, which was worked out by epidermal growth factor (EGF) stimulation in HEK cells, is also relevant in hippocampal neurons following activation with nerve growth factor (NGF), brain derived neurotrophic factor (BDNF) or insulin-like growth factor (IGF-1)^34^. In nociceptive neurons, TRPA1 is translocated to the plasma membrane when activated^35^ and similar mechanisms were described for other TRP channels though mostly in heterologous expression systems.

In summary, our results showing exaggerated FceRI- evoked calcium elevations in peritoneal mast cells from TRPM4−/− mice depict an important role of TRPM4 cation channels as a limiting regulators for antigen evoked calcium rise in connective tissue type mast cells. Lack of this regulatory mechanism might be responsible for the accelerated anaphylactic response in a passive cutaneous anaphylaxis test in TRPM4 deficient mice^8^. In addition, the antigen evoked translocation of TRPM4 proteins to the plasma membrane can explain the latency in development of TRPM4-mediated cation currents in mast cells,suggesting that TRPM4 protects against sustained calcium elevation following mast cell stimulation while permitting an initially sufficient Ca^2+^ -influx thereby limiting exaggerated persistent mast cell activation that triggers inflammatory and allergic reactions.

## Methods

### Animals

The generation of *Trpm4*−/− mice used in this study was previously described^8^. Experiments were performed on adult TRPM4-deficient mice that were backcrossed for at least 6 generations into the 129/SvJ background. Age matched 129/SvJ mice were used as controls for Ca^2+^ imaging and electrophysiological recordings. Mice were housed in an essentially specific pathogen–free environment with a 12-hour light/12-hour dark cycle and allowed water and standard food ad libitum. All methods were performed in accordance with German legislation on the protection of animals and were approved by the local ethics committee of the Universities of Saarland (Kreispolizeibehörde des Saarpfalz-Kreises, reference number K110/180-07) and Heidelberg (Regierungpräsidium Kalrsruhe, T-36-12, T-8-15).

### Peritoneal mast cell (PCMC) culture

For the isolation of PCMCs, peritoneal cells were gained by washing the peritoneal cavity (peritoneal lavage) of 3–4 mice with PCMC medium (450 ml RPMI 1640, 50 ml FCS, 0.5 ml 0.02% α-Monothiolglycerol in RPMI and 5 ml PenStrep) and culturing the cells with 10 ng/ml IL-3 and 30 ng/ml SCF as described^12^. On day 2 after cultivation all non-adherent cells were discarded. Cells were splitted on day 14 and 21 and used for experiments from day 14 to 28. Flow cytometry anaylsis, which was performed essentially as described^8^ to detect FcεRI and c-Kit in the plasma membrane, identified 98.5 to 99.5% of the cultured cells as mast cells.

### RT-PCR

BMMC cells were isolated and cultured as described^8^. For RNA isolation BMMCs or PCMCs were dissolved in buffer RLT (Qiagen). Total RNA was extracted using the Qiagen RNeasy Mini Kit. The eluted RNA (100 ng for BMMC and PCMC) was used for one step reverse transcription-PCR (RT-PCR, Invitrogen). The following intron-spanning primers were used for amplification of Trpm4, Trpm5 and Hprt fragments, respectively: Trpm4: 5′-TCTTCACACTGCGCCTGCTG-3′ and 5′-GTCGGTAGAAGACCCTGCGC-3′ resulting in a 246 bp fragment; Trpm5: 5′-AGTCACCTGTAGAATGGTGC-3′ and 5′-GAATGTGTAGCTGAACATGGC-3′ resulting in a 484 bp fragment; Hprt: 5′-GCTCGAGATGTCATGAAGG-3′ and 5′-AGTTGAGAGATCATCTCCACC-3′ (UW 637 und 638) resulting in a 225 bp fragment. In control experiments, total RNA from mouse brain (20 ng, Trpm4) or mouse tongue (100 ng, Trpm5) isolated using PeqGold kit was used.

### Calcium imaging

PCMCs were isolated and cultured from wild type (129SvJ) and Trpm4−/− mice^8^. Intracellular Ca^2+^ concentration was measured on day 14 of PCMC culture. Cells were loaded with 2 μM Fura-2, acetoxymethyl ester for 20–30 minutes at 37 °C in standard extracellular solution ([mM] NaCl 150, KCl 6, MgCl2 1, CaCl2 1.5, HEPES 10, glucose 10). Cells were incubated with anti-DNP IgE (300 ng/ml) over night, plated on Poly-L lysine (0.001%) coated cover slips and fluorescence at 510 nanometer was measured during excitation at 340 nm and 380 nm. [Ca^2+^]_i_ was measured with a monochromator-based imaging system consisting of a Polychrome V monochromator (TILL Photonics, Germany) and a charge-coupled device camera (Rolera EM-C2TM, QImaging, Canada) connected to an Axio Observer-A1 inverted microscope (Zeiss Jena, Germany). The monochromator and camera were controlled by the ZENII software (Zeiss). After correction for the background fluorescence signals, the ratio of the fluorescence at both excitation wavelengths (*F*_340_/*F*_380_) was monitored and analyzed in Origin software (Northampton, USA).

### Virus generation

Semliki Forest Virus (SFV): TRPM4-EYFP SFV was generated by subcloning a Myc-TRPM4-EYFP sequence including a Kozak sequence into the multiple cloning site of a modified pSFV1-plasmid from Invitrogen. The modified SFV1-plasmid contained a SwaI-sequence downstream of the Trpm4 cDNA to allow for linearisation. Following linearisation, the TRPM4-EYFP-SFV plasmid and the SFV-helper plasmid, that contains the structural proteins of the SFV-virus, were transcribed into RNA by reverse transcription. Both RNAs were transfected into BHK cells by electroporation (2 × 140V à 25 ms) using a GenePulser Xcell electroporation system (Biorad). TRPM4-EYFP SFV particles were obtained from the supernatant of the transfected BHK cells 24 h after electroporation and frozen in aliquots. For SFV activation 450 μl SFV suspension was thawed and 450 μl Optimem containing 0.2%BSA and 100 μl chymotrypsin (2 mg/ml in HBSS) was added and incubated for 40 min at room temperature. The chemical reaction was stopped by 110 μl Aprotinin (6 mg/ml in HBSS). SFV suspension was then used for transduction experiments.

Lentivirus: the Myc-TRPM4-EYFP sequence was subcloned into a PGK-promotor containing pRRL lentiviral vector. Lentiviral particels were generated according to previously described protocols^30^ following transfection of HEK293 cells with four different plasmids using the calcium phosphate method. Lentiviruses were obtained from the supernatent and concentrated as described^36^.

### Viral cell transduction

SFV: For PCMC transduction TRPM4-EYFP-SFV were added to PCMC cultures between day 14 to 28 at a MOI of 17 for 90 min. Cells were centrifugated at 140 g and cultured in PCMC medium for another 8.5 hours after which confocal microscopy was performed.

Lentivirus: For lentiviral transduction PCMCs were cultured in medium containing TRPM4-EYFP lentivirus (MOI = 2) and 0,9 μg/ml Polybrene from day 2 of peritoneal cell preparation to day 9 at which the cells were splitted. TRPM4-EYFP expressing PCMCs were used for experiments from day 13 to 28.

HEK-TRPM4-EYFP-LV cells: HEK293-T cells were counted and seeded 12 hours prior to TRPM4-eYFP lentivirus addition. Lentiviral particles were added with a MOI of 20 in new medium. After 24 h, the volume of medium was increased to 2.5-fold. After 48 h hours, cells were microscopically analyzed for transduction efficiency and split. After one week, flow cytometry analysis was performed (transduction efficiency: 91.5%).

### Fluorescence microscopy

For microscopic analysis PCMCs were allowed to adhere to coverslides which were pretreated with 0.001% Poly-L lysine (PLL) in PBS for 60 min, washed with PBS and air dryed at room temperature. For experiments employing DNP-stimulation PCMC were incubated for 15 min on PLL-treated coverslides, and DNP was added before (Fig. 3) or during microscopy (Fig. 4). In initial experiments a higher PLL concentration (0.01% PLL) was applied with subsequent evaporation on a heating plate at 50 °C.

Epifluorescence microscopy was done with a Zeiss Z1 fluorescence microscope with a 63x objective (Plan Apochromat/1,4 NA Oil DIC III). TRPM4-EYFP was detected with a HC Basic YFP filter (AHF Analysentechnik, Tübingen, Germany).

For confocal microscopy a spinning disk QLC-100 (VisiTech Ltd, UK) scan head attached to an upright Nikon E600 miscroscope was used (Emission-filter: 390/482/563/640 nm BrightLine^®^ quad-band bandpass filter Dichroid-filter: 405/488/568/647 nm Yokogawa dichroic beamsplitter). Fluorecence was detected with a CCD-camera (OrcaER, Hamamatsu, Japan). Experimentds were controlled by VoxCell Scan software (VisiTech Ltd., UK). TRPM4-EYFP was excitated with a 488 nm laser (DPSS, Sapphire 488-30, Coherent, USA). The cell mask deep red plasma membrane stain (1,6 μg/ml) was applied for 5 minutes at room temperature. Afterwards cells were washed with Krebs-Henseleit buffer and excitated with a 635 nm laser line (DPSS, 85-YCA-015, Melles Griot, USA). During the microscopy, cells were suspended in Krebs-Henseleit Buffer. For adhesion protocols with 0.001% PLL a 100x water objective NA 1,1 (2,5 DIC, H/N2, Nikon) was used in an inverted microscopy chamber. For adhesion protocols with 0.01% PLL a 100x oil objective NA 1,4 (Apo, Ph 3, Nikon) was used.

For colocalisation analysis regarding plasma membranous TRPM4-EYFP expression the Manders coefficient of plasma membrane dye signal overlapping the TRPM4-EYFP signal (M_1_) after automatic threshold correction was calculated using the JACoP v2.0 Plugin in ImageJ^37^. For the analysis of fluorescence intensity distribution before and after DNP stimulation, 16 concentric lines were drawn through the cell as shown in Figs 3C and 4C. Fluorescence intensity distribution was then analysed along the 16 lines using multi plot analysis in ImageJ. Plasma membranous TRPM4-EYFP localisation was attributed to the average of both peaks of the x-y fluorescence intensity profile corresponding to the cell borders.

HEK-TRPM4-EYFP-LV cells: Plasma membrane colocalisation of TRPM4-EYFP was determined by a confocal microscopy system (TCS SP5 X, Leica, Germany) with an upright microscope (STP 6000 CFT, Leica) using a 63x oil objective (HCX PL Apo 63x/1.4–0.6 oil CS, 506188, Leica). Microscope settings and image acquisition were controlled by LAS AF Lite software (Leica, Germany). HEK-TRPM4-EYFP cells were seeded on coverslips 24 hours before analysis. Living HEK-TRPM4-EYFP cells were stained with CellMaskTM (C10046, Thermo Fisher) deep red plasma membrane stain (5 μg/ml) for 10 min at 37 °C followed by gently washing with DPBS (Thermo Fisher). TRPM4-EYFP was excited with a 488 nm argon laser (emission range 520–620 nm), CellMaskTM was excited with a 633 nm HeNe Laser (emission range 693–800 nm).

### Electrophysiological experiments

Currents were measured using EPC- 10 (HEKA Elektronik) “patch-clamp” amplifier in the whole-cell configuration. The ramp protocol consisted of a 400-ms ramp from −100 mV to +100 mV (holding potential is 0 mV) applied at 0.5 Hz. Recordings were started immediately after achievement of whole-cell configuration. Experiments were done at 22–25 °C. Solutions: The standard extracellular solution for patch-clamp contained 135 mM NaCl, 6 mM KCl, 2 mM CaCl_2_, 1.2 mM MgCl_2_, 12 mM glucose, 10 mM HEPES, pH 7.4, with NaOH. The pipette solution for whole-cell measurements contained potassium glutamate 135 mM, 20 mM NaCl, 0.2 mM MgATP, 0.3 mM GTP, 1 mM MgCl_2_, 10 mM HEPES, 5 mM HEDTA, 3.6 mM CaCl_2_ pH 7.2, with KOH. All chemicals were purchased from Sigma.

### Statistics

For statistical analysis IBM SPSS software was used. For the analysis of an observed frequency distribution the Fisher’s exact test was used. For the analysis of the edging peak fluorescence intensity in PCMCs without and after FcεRI-stimulation (SFV transduction) the t-test for independent samples was used after having confirmed normal distribution of all values in each group with the Kolmogorow-Smirnow test. For fluorescence intensity analysis before and after cell stimulation in the same cell (lentiviral transduction) the t-test for dependent samples was used after confirmation of a normal distribution of the determined differences of the peak fluorescence intensity values using the Shapiro-Wilk test. Each cell was assigned the average edging fluorescence value before and after cell stimulation, respectively.

## Additional Information

**How to cite this article**: Rixecker, T. *et al*. TRPM4-mediated control of FcεRI-evoked Ca^2+^ elevation comprises enhanced plasmalemmal trafficking of TRPM4 channels in connective tissue type mast cells. *Sci. Rep*. **6**, 32981; doi: 10.1038/srep32981 (2016).

## Supplementary Material

Supplementary Information
