# Supplementary material for: TRPM4-mediated control of FcεRI-evoked Ca2+ elevation comprises enhanced plasmalemmal trafficking of TRPM4 channels in connective tissue type mast cells
Source: Sci Rep. 2016 Sep 14;6:32981. doi: 10.1038/srep32981 (PMC5021962; doi:10.1038/srep32981)

Supplementary Information to

TRPM4-mediated control of FcεRI-evoked Ca^{2+} elevation comprises enhanced plasmalemmal trafficking of TRPM4 channels in connective tissue type mast cells

Torben Rixecker[1], Ilka Mathar[1], Rebekka Medert[1], Stefanie Mannebach[2], Alexander Pfeifer[4], Peter Lipp[3], Volodymyr Tsvilovskyy[1], Marc Freichel[1]

1 Pharmakologisches Institut, Ruprecht-Karls-Universität Heidelberg, 69120 Heidelberg, Germany. 2 Experimentelle und Klinische Pharmakologie und Toxikologie, 3 Institut für Molekulare Zellbiologie Universität des Saarlandes, 66421 Homburg, Germany. 4 Institute of Pharmacology and Toxicology, University Hospital Bonn, University of Bonn, 53127 Bonn, Germany.

Correspondence to: Marc Freichel, INF-366, D-69120 Heidelberg, Germany, Tel.: +49-6221-54-86861, Fax: +49-6221-54-8644 (marc.freichel@pharma.uni-heidelberg.de)

Legend to Suppl. Fig.1: Transduction efficacy of TRPM4-EYFP SFV and TRPM4-EYFP lentivirus in PCMC and mast cell morphology before and after cell transduction with SFV

A: Bright field and fluorescence images of W PCMCs transduced with TRPM4-EYFP SFV. WT PCMC were transduced with TRPM4-EYFP-SFV (MOI = 17) and incubated for 10h prior to fluorescence microscopy on PLL coated (0.01%) coverslides for 15min. Transduction efficacy was 9,3% (n = 2 transduction experiments). Shown are bright field and YFP fluorescence images recorded using a QLC-100 confocal scan head and a 100x oil objective.

B: Mast cell morphology before and after cell transduction with TRPM4-EYFP SFV shown by DIC-Images otained with a Leica TCS SP5 II confocal microscope and a 63x oil objective.

C: Bright field and YFP fluorescence of WT PCMCs transduced with TRPM4-EYFP lentivirus. WT PCMCs were transduced with TRPM4-EYF -lentivirus (MOI = 2, Polybrene = 0,9µg/ml) and incubated for 13d before fluorescence microscopy on PLL (0,01%) coated coverslides. Transduction efficacy was 3,4% (n = 2 transduction experiments). Shown are bright field and epifluorescence images made on a Zeiss Z1 microscope with a 63x objective and a HC Basic YFP Filter.

Legend to Suppl. Fig. 2: TRPM4-EYFP expression in PCMC using TRPM4-EYFP SFV after incubation on low or high concentrated Poly-L-Lysin coatings

(A) Representative examples of PCMCs with substantial plasma membranous localization (pm) of TRPM4-EYFP and with non-plasma membranous (n-pm) TRPM4-EYFP localization after incubation on low (0.001%, left panel) or high concentrated (0.01% middle and right panel) Poly-L-Lysin coatings at the indicated time points. **(B)** Bar graphs show the number of cells assigned to whether TRPM4-YFP could be identified in the plasma membrane (pm) or not (n-pm); * $p < 0,05$ (Fisher's exact test, n = 6 (0,001% PLL), n = 3 (0,01% PLL, 15min). Microscopy was performed using an Nikon E600 microscope with a QLC-100 scan head and a 100x oil objective. Fluorescence was measured 10h after cell transduction with TRPM4-EYFP-SFV. (C) Representative PCMC plated on a PLL (0,01%)-coated coverslip with substantial plasma membranous localization (pm) of TRPM4-EYFP. Visualisation of the plasma membrane using the cell mask deep red stain (Invitrogen) indicates significant plas-

ma membrane extrusions. Manders coefficient M1 of the co-localisation analysis of TRPM4-EYFP and the cell mask deep red stain is indicated.

Suppl. Figure 1

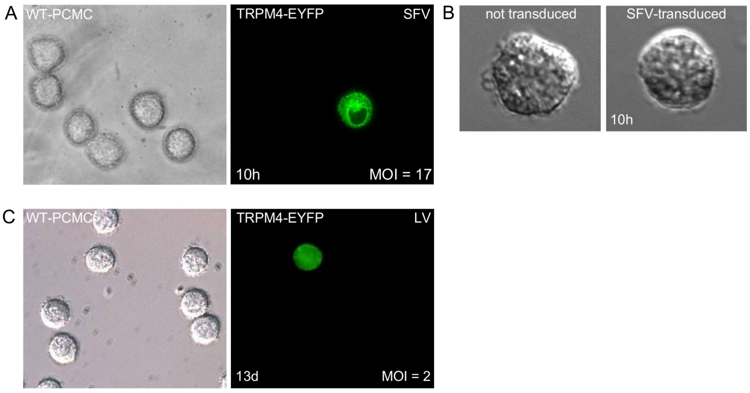

Suppl. Figure 2

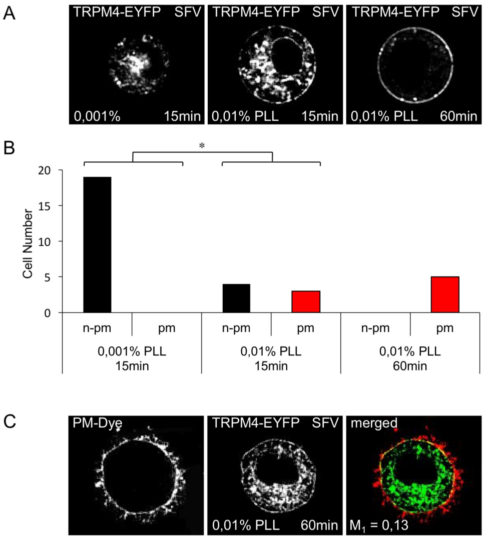

Supplement: Supplementary Information [file srep32981-s1.pdf]
